# Supplementary material for: System-wide identification and prioritization of enzyme substrates by thermal analysis
Source: Nat Commun. 2021 Feb 26;12:1296. doi: 10.1038/s41467-021-21540-6 (PMC7910609; doi:10.1038/s41467-021-21540-6)
Supplement: Supplementary file 1 — Supplementary Information [file 41467_2021_21540_MOESM1_ESM.pdf]

SUPPLEMENTARY INFORMATION

System-wide Identification and prioritization of Enzyme Substrates by Thermal Analysis

Saei at al.

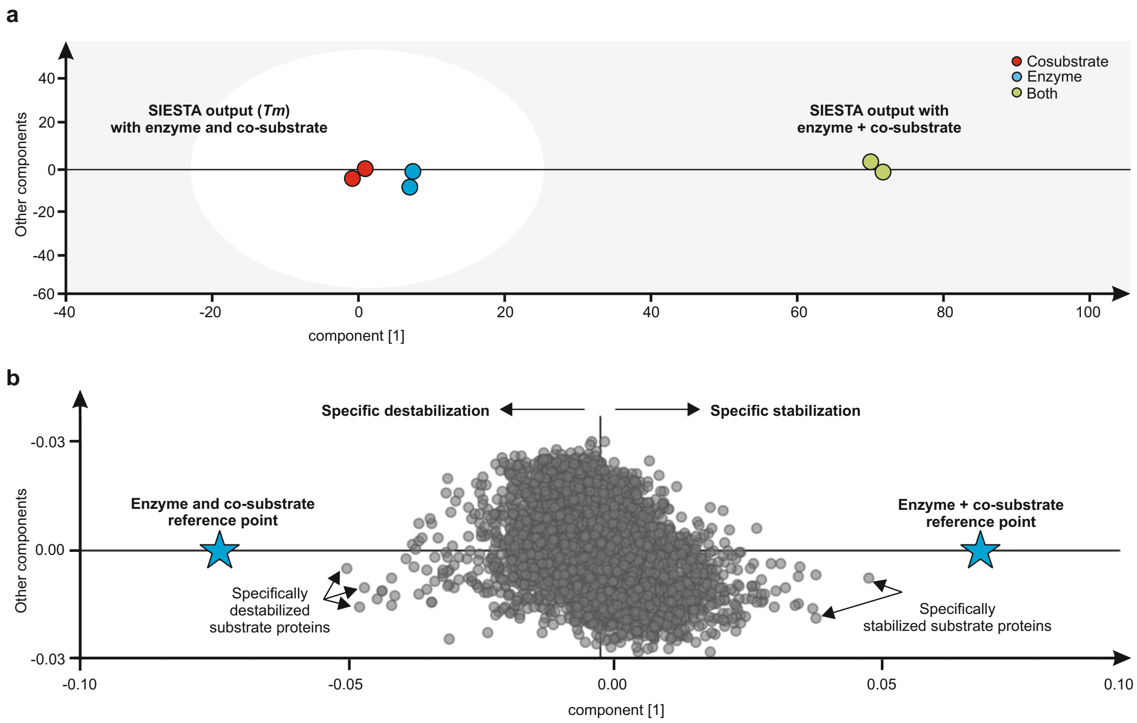

Supplementary Fig. 1. OPLS-DA modeling scheme and interpretation. a A generalized OPLS-DA model contrasting the SIESTA output (*Tm*s for all the proteins) in response to enzyme+co-substrate vs. all other treatments (enzyme- and co-substrate-treated samples). **b** The OPLS-DA loading or score scatter plot demonstrating proteins most contributing to class separation. The specific substrates can therefore be found on the x extremities (*Tm*, melting temperature).

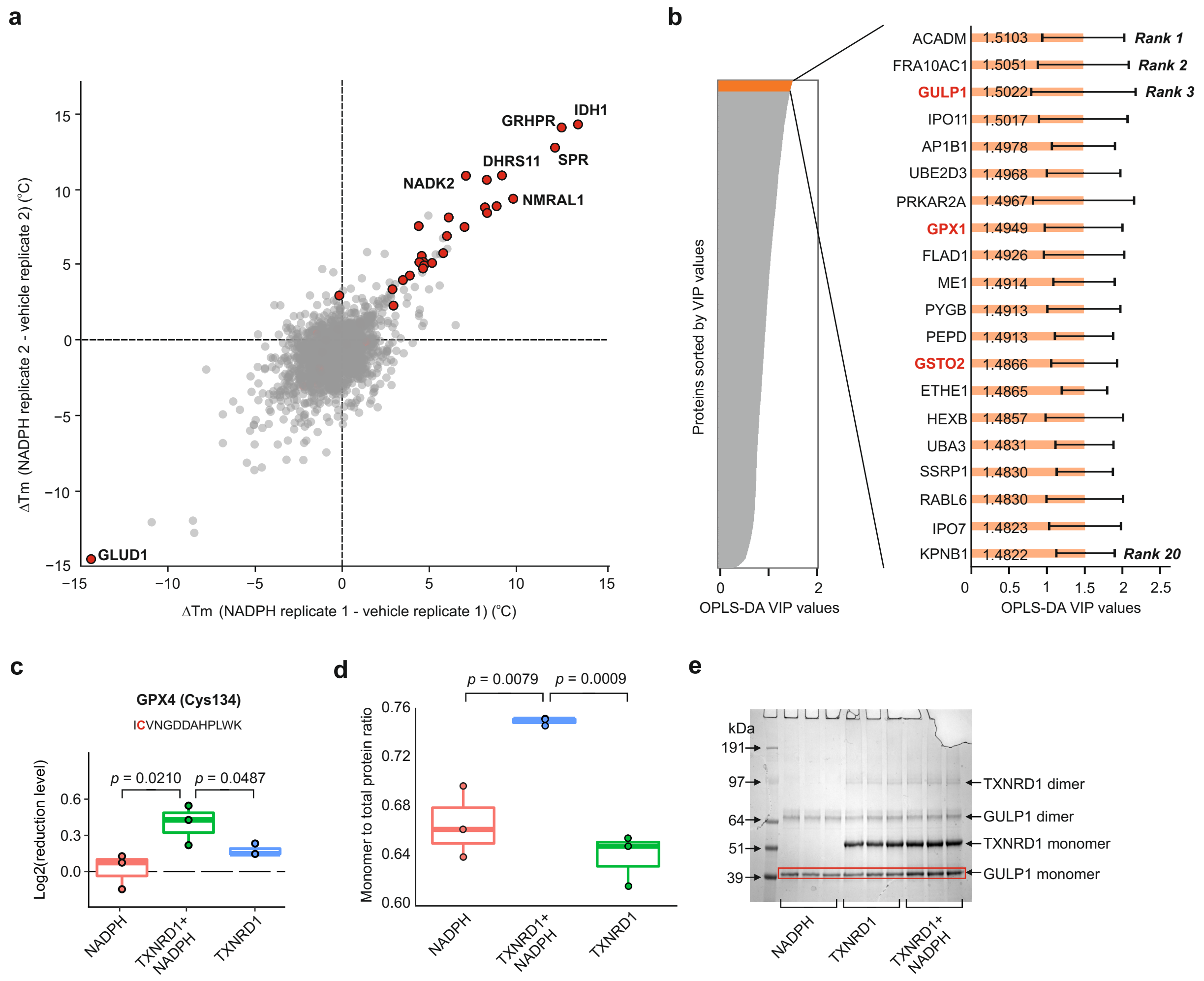

Supplementary Fig. 2. Confirmation of TXNRD1 substrates by orthogonal assays. a, The reproducibility between the replicates for identification of NADPH binding proteins. Scatterplot of protein *Tm* differences upon addition of NADPH in lysate. Known NADPH binding proteins from UniProt are shown in red. **b,** The VIP values and confidence intervals extracted from the OPLS-DA model in **Fig. 2d** (whiskers represent 95% confidence intervals). The proteins are ranked by the highest VIP values, which for top proteins are all positive within the confidence interval. **c,** The reduction level of GPX4 Cys134 in the presence of TXNRD1, NADPH or both (*n*=3 independent biological replicates; one-sided Student t-test for the redox experiment). **d-e,** The GULP1 monomer to total monomer+dimer levels upon treatment with TXNRD1, NADPH or both (*n*=3 independent biological replicates, two-sided

Student t-test; the samples derive from the same experiment and were processed in parallel) (Boxplots: Center line - median; box limits contain 50% of data; upper and lower quartiles, 75 and 25%; maximum - greatest value excluding outliers; minimum - least value excluding outliers; outliers - more than 1.5 times of the upper and lower quartiles) (OPLS-DA, orthogonal partial least squares-discriminant analysis; *Tm*, melting temperature; VIP, variable influence on projection). Source data are available as a Source Data file.

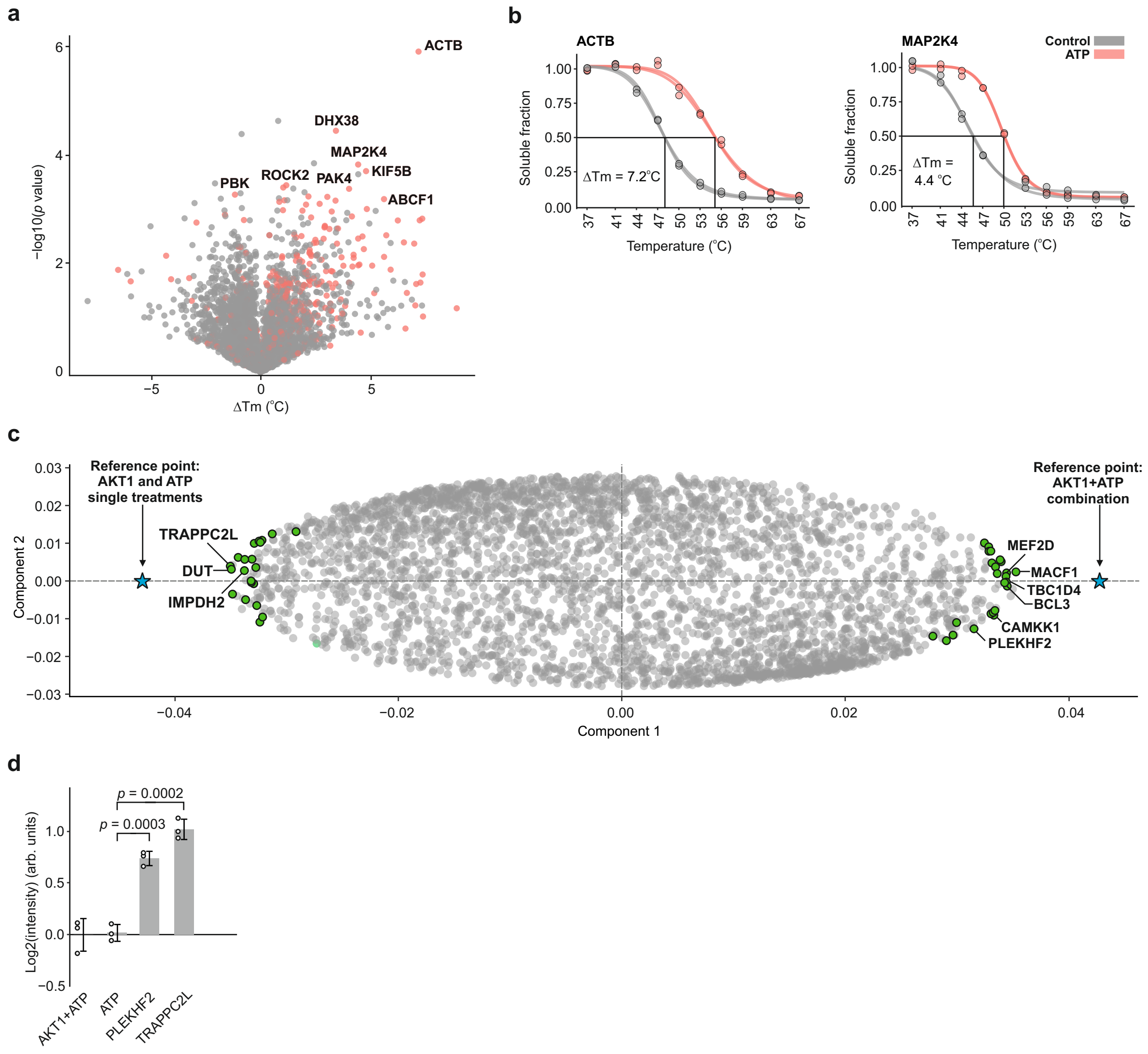

Supplementary Fig. 3. Analysis of SIESTA experiment for AKT1. a, Proteins shifting with 500 µM ATP in HELA cell lysate (known ATP binding proteins from UniProt are shown in red) (*n*=2 independent biological replicates; two-sided Student t-test; no adjustment for multiple comparisons was performed). **b,** Representative proteins shifting with 500 µM ATP. **c,** The OPLS-DA model contrasting the T_m in AKT1+ATP treatment against AKT1 and ATP alone. Proteins shown in green are those identified as substrates in **Fig. 3a**. **d,** The relative levels of phosphate release as measured with Phosphoprotein Phosphate Estimation assay (*n*=3 and data presented as mean±SD; two-sided Student t-test). Source data are available as a Source Data file.

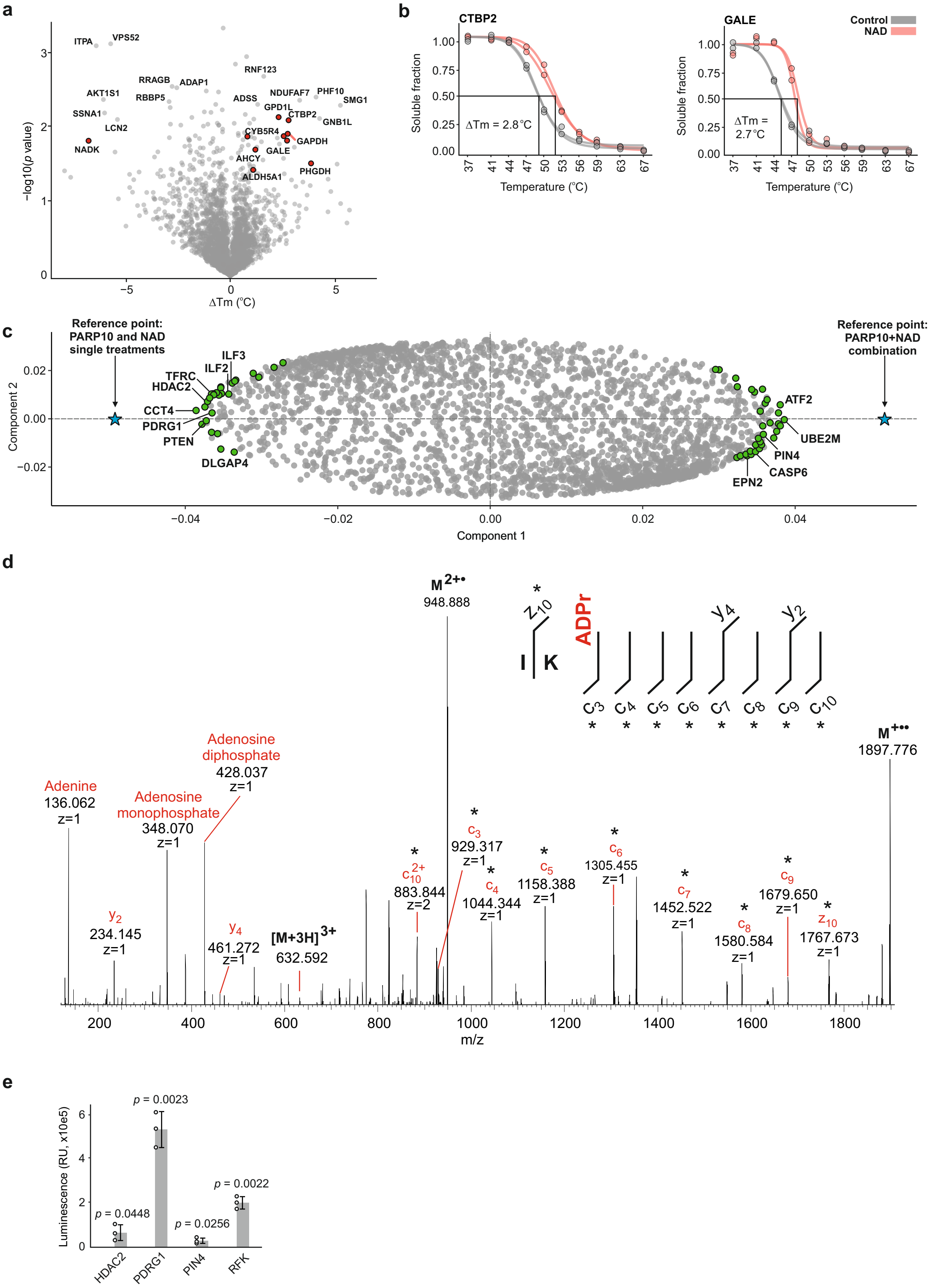

a
ITPA
VPS52
RNF123
RRAGB
ADAP1
AKT1S1
RBBP5
NDUFAF7
PHF10
SMG1
ADSS
GPD1L
SSNA1
CTBP2
GNB1L
LCN2
CYB5R4
GAPDH
NADK
GALE
AHCY
PHGDH
ALDH5A1
−log10(p value)
ΔTm (°C)
b
CTBP2
GALE
Control
NAD
Soluble fraction
ΔTm = 2.8°C
ΔTm = 2.7°C
Temperature (°C)
c
Reference point: PARP10 and NAD single treatments
Reference point: PARP10+NAD combination
ILF3
ILF2
TFRC
HDAC2
CCT4
PDRG1
PTEN
DLGAP4
ATF2
UBE2M
PIN4
CASP6
EPN2
Component 2
Component 1
d
M 2+• 948.888
ADPr
I K
M+•• 1897.776
Adenine 136.062 z=1
Adenosine diphosphate 428.037 z=1
Adenosine monophosphate 348.070 z=1
y2 234.145 z=1
y4 461.272 z=1
[M+3H]3+ 632.592
c10 2+ 883.844 z=2
c3 929.317 z=1
c4 1044.344 z=1
c5 1158.388 z=1
c6 1305.455 z=1
c7 1452.522 z=1
c8 1580.584 z=1
c9 1679.650 z=1
z10 1767.673 z=1
m/z
e
Luminescence (RU, x10e5)
p = 0.0023
p = 0.0022
p = 0.0448
p = 0.0256
HDAC2
PDRG1
PIN4
RFK

Supplementary Fig. 4. Analysis of SIESTA experiment for PARP10. a) A volcano plot with ΔTm of NAD vs. control. The outliers are putative NAD binding proteins. Known NAD binders in UniProt database are shown in red circles (*n*=2 independent biological replicates; two-sided Student t-test; no adjustment for multiple comparisons was performed). **b,** Representative melting curves of NAD sensor CTBP2 and the NAD binding protein GALE. **c,** Loadings of OPLS-DA model contrasting the PARP10+NAD T_m vs. those in all other treatments singled out potential substrates (green circles). Proteins shown in green are those identified as substrates in **Fig. 4a**. **d,** Targeted ETD MS/MS of a RFK peptide revealed mono-ADP-ribosylation on glutamic acid residue (the site with the highest sequence-fitting score). The fragments carrying the modification are marked with an asterisk (M = molecular ion). **e,** The mono-ADP-ribosylation of HDAC2, PIN4, PDRG1 and RFK was confirmed upon incubation with PARP10 catalytic domain and NAD (*n*=3 and data presented as mean±SD; one-sided Student t-test). Source data are available as a Source Data file.

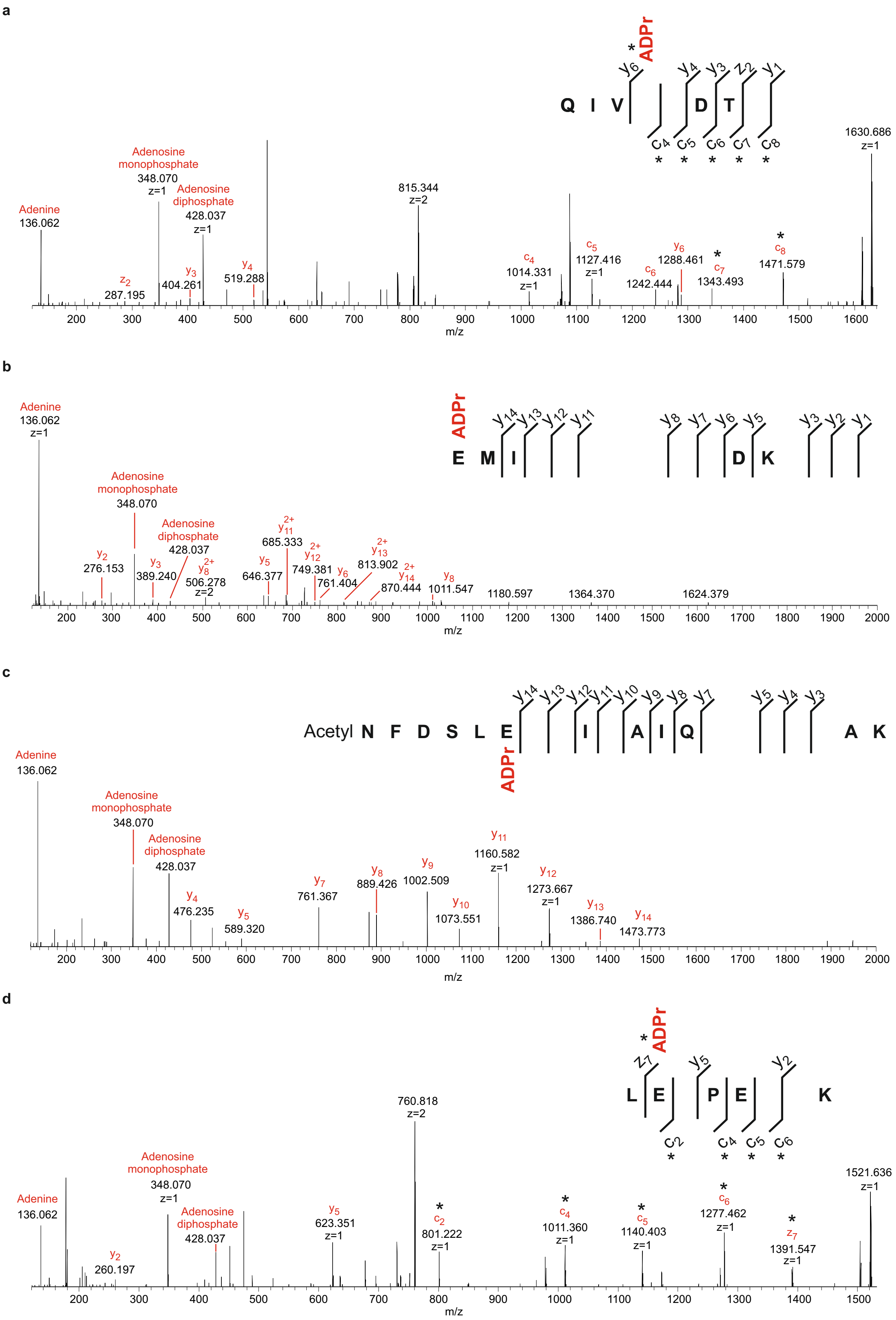
a
ADPr
Q I V D T
y6 y4 y3 z2 y1
c4 c5 c6 c7 c8
Adenine
136.062
Adenosine monophosphate
348.070
z=1
Adenosine diphosphate
428.037
z=1
z2
287.195
y3
404.261
y4
519.288
815.344
z=2
c4
1014.331
z=1
c5
1127.416
z=1
c6
1242.444
y6
1288.461
c7
1343.493
c8
1471.579
1630.686
z=1
m/z
b
ADPr
E M I D K
y14 y13 y12 y11 y8 y7 y6 y5 y3 y2 y1
Adenine
136.062
z=1
Adenosine monophosphate
348.070
Adenosine diphosphate
428.037
y2
276.153
y3
389.240
y8 2+
506.278
z=2
y5
646.377
y11 2+
685.333
y12 2+
749.381
y6
761.404
y13 2+
813.902
y14 2+
870.444
y8
1011.547
1180.597
1364.370
1624.379
m/z
c
Acetyl N F D S L E I A I Q A K
ADPr
y14 y13 y12 y11 y10 y9 y8 y7 y5 y4 y3
Adenine
136.062
Adenosine monophosphate
348.070
Adenosine diphosphate
428.037
y4
476.235
y5
589.320
y7
761.367
y8
889.426
y9
1002.509
y10
1073.551
y11
1160.582
z=1
y12
1273.667
z=1
y13
1386.740
y14
1473.773
m/z
d
ADPr
L E P E K
z7 y5 y2
c2 c4 c5 c6
Adenine
136.062
Adenosine monophosphate
348.070
z=1
Adenosine diphosphate
428.037
y2
260.197
y5
623.351
z=1
760.818
z=2
c2
801.222
z=1
c4
1011.360
z=1
c5
1140.403
z=1
c6
1277.462
z=1
z7
1391.547
z=1
1521.636
z=1
m/z

Supplementary Fig. 5. Validation of ADP-ribosylation on multiple sites for PDRG1 and RFK. a-b) ADP-ribosylation of PDRG1 and **c-d**) RFK on two sites. Note the presence of signature ions of adenine (*m/z* 136.062), adenosine monophosphate (*m/z* 348.070) and adenosine diphosphate (*m/z* 428.037). The fragments carrying the modification are marked with an asterisk.

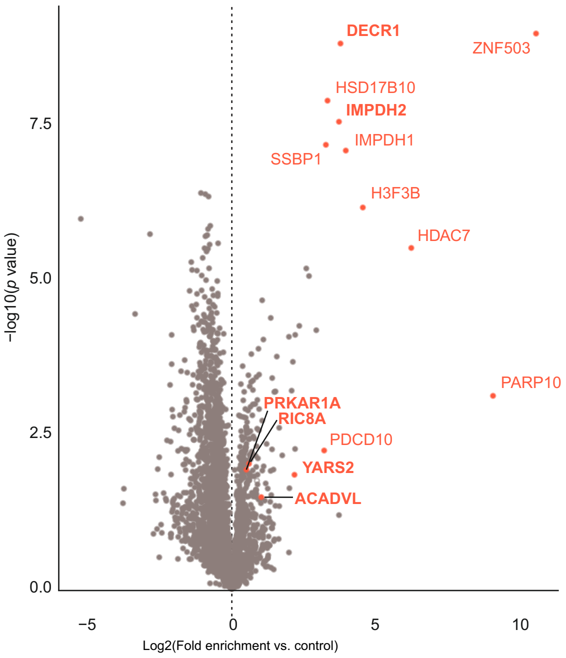

Supplementary Figure 6. PARP10 pulldown in HCT116 cell lysate. The outliers are enriched in the pulldown experiment. The proteins in bold are those identified in SIESTA as PARP10 interactors and show a significant change in stability (*n*=4 independent biological replicates; two-sided Student t-test; no adjustment for multiple comparisons was performed). Source data are available as a Source Data file.

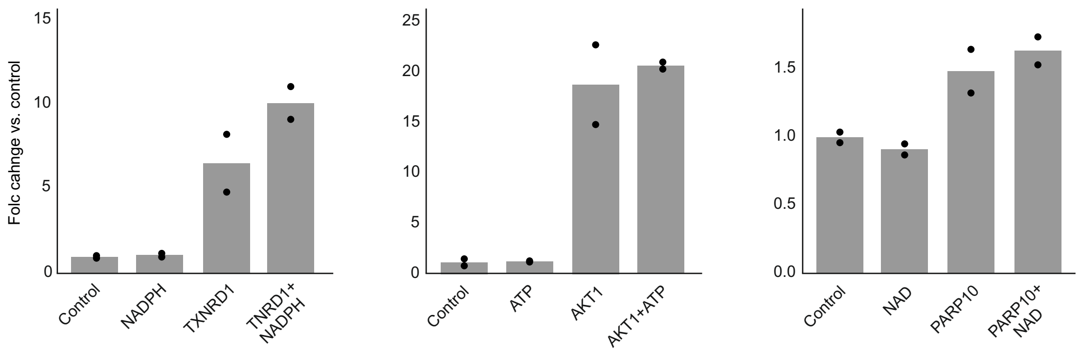

Supplementary Figure 7. Added enzyme ratio in SIESTA experiments. The ratio of enzyme in samples treated with enzyme compared to untreated or co-substrate-treated lysate in each SIESTA experiment (*n*=2 independent biological replicates; data presented as mean). Source data are available as a Source Data file.

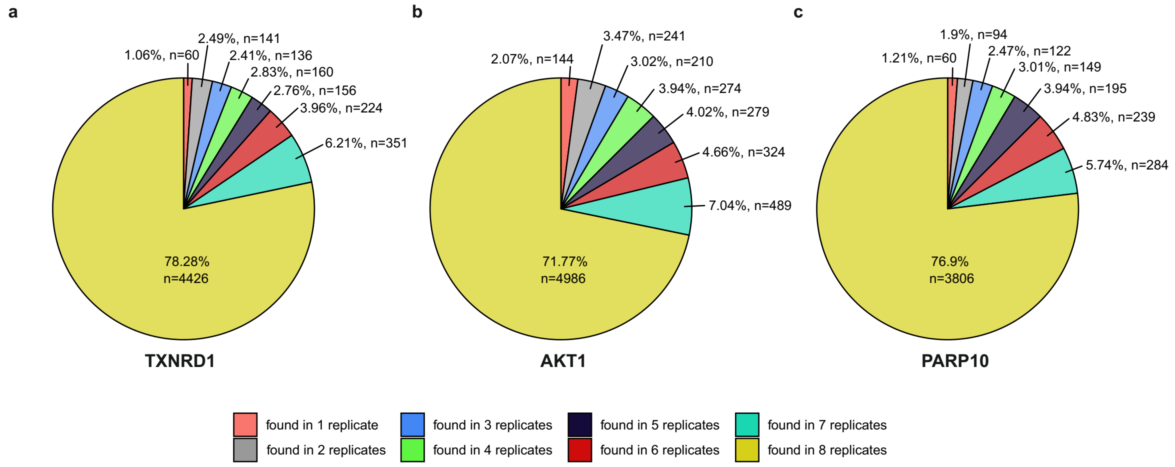

Supplementary Figure 8. The percentage and number of proteins with at least 2 peptides and no missing values across all replicates in each SIESTA experiment. The useable fraction is shown in mustard. Source data are available as a Source Data file.

Supplementary Tables

Supplementary Table 1. The PARP10-mediated mono-ADP-ribosylation sites of PDRG1 and RFK. The modified amino acids are shown in bold red in the peptide sequence.

Protein	Start	End	Mascot Score	Peptide (site of modification)
**PDRG1**	29	37	1	R.QIV**D**LDTKR.N + ADP-Ribosyl (D)
	75	90	12	K.**E**MIEKDQDHLDKEIEK.L + ADP-Ribosyl (E)
	102	112	12	R.LFEAQGKP**E**LK.G + ADP-Ribosyl (E)
**RFK**	108	127	61	K.NFDSL**E**SLISAIQGDIEEAK.K + Acetyl (N-term); ADP-Ribosyl (E)
	130	137	14	R.L**E**LPEHLK.I + ADP-Ribosyl (E)
	130	148	14	R.L**E**LPEHLKIKEDNFFQVSK.S + Acetyl (N-term); ADP-Ribosyl (E)
	138	148	39	K.IK**E**DNFFQVSK.S + ADP-Ribosyl (E)

Supplementary Table 2. The recombinant proteins used in this study

Protein	Catalogue number	Source
Protein kinase B (AKT1) catalytic domain	01-401-20N	Carna Biosciences (Japan)
RFK	ab89009	Abcam
PDRG1	PRO-007	ProSpec
Caspase-6	ALX-201-060-U100	Enzo
HDAC2	BML-SE533-0050	Enzo
GSTO1	NBP1-37093	Novus
GSTO2	ab124576	Abcam
PRDX2	ab85331	Abcam
GULP1	ab140546	Abcam
PLEKHF2	PRO-1874	ProSpec
TRAPPC2L	PRO-1543	ProSpec

Supplementary Table 3. The LC-MS parameters used in each experiment as well as the number of quantified proteins

**Experiment**	**SIESTA TXNRD1**	**SIESTA PARP10**	**SIESTA AKT1**	**Redox experiment with iodoTMT**	**Phospho-proteomics**	**PARP10 pulldown**
**Cell line**	HCT-116	HCT-116	HELA	-	HELA	HCT-116
**Number of fractions per replicate**	8	8	24	-	8x2	1
**Instrument**	Fusion	Fusion	Q Exactive HF	Q Exactive Plus	Fusion	Fusion
**Total gradient time (min)**	120	160	95	120	110	240
**MS scan range (m/z)**	400-1600	400-1600	375-1500	375-1500	400-1600	350-1500
**HCD collision energy**	40	40	33	32	35	30
**Orbitrap resolution**	120,000	120,000	120,000	70,000	120,000	120,000
**MS^2 resolution**	60,000	60,000	60,000	35,000	60,000	30,000
**MS AGC target**	1e6	1e6	3e6	3e6	1.25e5	4e5
**MS^2 AGC target**	1e5	1e5	2e5	2e5	1.25e5	1e5
**MS maximum injection time (ms)**	50	50	100	120	Auto	50
**MS^2 maximum injection time (ms)**	105	105	120	120	Auto	60
**Isolation window**	0.7	0.7	1.6	1.2	1.6	1.2
**Dynamic exclusion**	60	60	45	45	60	60
**Number of quantified proteins**	5864	5194	7179	-	-	-
