# Supplementary material for: System-wide identification and prioritization of enzyme substrates by thermal analysis
Source: Nat Commun. 2021 Feb 26;12:1296. doi: 10.1038/s41467-021-21540-6 (PMC7910609; doi:10.1038/s41467-021-21540-6)
Supplement: Supplementary file 3 — Description of Additional Supplementary Files [file 41467_2021_21540_MOESM3_ESM.docx]

**Description of Supplementary Files**

**File Name: Supplementary Data 1**

**Description:** Sheet 1, The processed data for TXNRD1 SIESTA experiment for all the replicates; Sheet 2, NADPH binding proteins (twosided Student t-test; no adjustment for multiple comparisons was performed); Sheet 3, the identified substrates for TXNRD1 (two-tailed Student t-test; no adjustment for multiple comparisons was performed); Sheet 4, OPLS-DA scores contrasting TXNRD1+NADPH vs. TXNRD1 and NADPH single treatments.

**File Name: Supplementary Data 2**

**Description:** The normalized ratio of the abundances of peptide from the candidate substrate proteins when the following molecules are added to cell lysate: TXNRD1, NADPH or TXNRD1+NADPH.

**File Name: Supplementary Data 3**

**Description:** **Sheet 1:** The processed data for AKT1 SIESTA experiment for all replicates; Sheet 2: ATP binding proteins (two-tailed Student t-test; no adjustment for multiple comparisons was performed); Sheet 3: the identified substrates for AKT1 (two-tailed Student t-test; no adjustment for multiple comparisons was performed); Sheet 4: OPLS-DA scores contrasting AKT1+ATP vs. single treatments with AKT1 and ATP.

**File Name: Supplementary Data 4**

**Description:** Phosphoproteomics analysis of HELA cells treated with 2.5 and 10 µM AKT1/2 inhibitor and ipatasertib (two-tailed Student ttest; no adjustment for multiple comparisons was performed).

**File Name: Supplementary Data 5**

**Description:** Sheet 1: The processed data for PARP10 SIESTA experiment for all replicates; Sheet 2: NAD binding proteins (two-tailed Student t-test; no adjustment for multiple comparisons was performed); Sheet 3: the identified substrates for PARP10 (two-tailed Student t-test; no adjustment for multiple comparisons was performed); Sheet 4: OPLS-DA scores contrasting PARP10+NAD vs. single treatments with PARP10 and NAD.

**File Name: Supplementary Data 6**

**Description:** The identified interactions with other proteins for TXNRD1, AKT1 and PARP10 (two-tailed Student t-test; no adjustment for multiple comparisons was performed).

**File Name: Supplementary Data 7**

**Description:** Proteins identified in the pulldown experiment identifying PARP10 interacting proteins in HCT116 cells (two-tailed Student t-test; no adjustment for multiple comparisons was performed).
