# Supplementary material for: System-wide identification and prioritization of enzyme substrates by thermal analysis
Source: Nat Commun. 2021 Feb 26;12:1296. doi: 10.1038/s41467-021-21540-6 (PMC7910609; doi:10.1038/s41467-021-21540-6)
Supplement: Supplementary file 11 — Reporting Summary [file 41467_2021_21540_MOESM11_ESM.pdf]

nature research

Corresponding author(s): Roman A. Zubarev

Last updated by author(s): Jan 21, 2021

Reporting Summary

Nature Research wishes to improve the reproducibility of the work that we publish. This form provides structure for consistency and transparency in reporting. For further information on Nature Research policies, see our Editorial Policies and the Editorial Policy Checklist.

Statistics

For all statistical analyses, confirm that the following items are present in the figure legend, table legend, main text, or Methods section.

n/a	Confirmed	
☐	☒	The exact sample size (n) for each experimental group/condition, given as a discrete number and unit of measurement
☐	☒	A statement on whether measurements were taken from distinct samples or whether the same sample was measured repeatedly
☐	☒	The statistical test(s) used AND whether they are one- or two-sided<br>*Only common tests should be described solely by name; describe more complex techniques in the Methods section.*
☒	☐	A description of all covariates tested
☒	☐	A description of any assumptions or corrections, such as tests of normality and adjustment for multiple comparisons
☐	☒	A full description of the statistical parameters including central tendency (e.g. means) or other basic estimates (e.g. regression coefficient) AND variation (e.g. standard deviation) or associated estimates of uncertainty (e.g. confidence intervals)
☐	☒	For null hypothesis testing, the test statistic (e.g. F, t, r) with confidence intervals, effect sizes, degrees of freedom and P value noted<br>*Give P values as exact values whenever suitable.*
☒	☐	For Bayesian analysis, information on the choice of priors and Markov chain Monte Carlo settings
☒	☐	For hierarchical and complex designs, identification of the appropriate level for tests and full reporting of outcomes
☒	☐	Estimates of effect sizes (e.g. Cohen's d, Pearson's r), indicating how they were calculated

Our web collection on statistics for biologists contains articles on many of the points above.

Software and code

Policy information about availability of computer code

Data collection	Thermo Xcalibur 4.0
Data analysis	Excel 2013, MaxQuant 1.5.6.5, SIMCA 15, R project versions 3.6 - 4.0, SIESTA package version 1 (in-house developed by the laboratory of Roman A Zubarev at Karolinska Institutet (https://github.com/RZlab/SIESTA), STRING version 10.5 (http://string-db.org), Mascot 2.5.1 (Matrix Science), RAWtoMGF v. 2.1.3, Quantity One 4.6.9

For manuscripts utilizing custom algorithms or software that are central to the research but not yet described in published literature, software must be made available to editors and reviewers. We strongly encourage code deposition in a community repository (e.g. GitHub). See the Nature Research guidelines for submitting code & software for further information.

Data

Policy information about availability of data

All manuscripts must include a data availability statement. This statement should provide the following information, where applicable:

- Accession codes, unique identifiers, or web links for publicly available datasets
- A list of figures that have associated raw data
- A description of any restrictions on data availability

The authors declare that all data supporting the findings of this study are available within the paper and its supplementary information files. All relevant data are available from the corresponding authors (A.A.S. and R.A.Z.). The mass spectrometry data that support the findings of this study have been deposited in ProteomeXchange Consortium (https://www.ebi.ac.uk/pride/) via the PRIDE partner repository with the dataset identifiers PXD010554 [http://www.ebi.ac.uk/pride/archive/projects/PXD010554] for PARP10 and TXNRD1 SIESTA, PXD014445 [http://www.ebi.ac.uk/pride/archive/projects/PXD014445] for AKT1 SIESTA, PXD021915 [http://www.ebi.ac.uk/pride/archive/projects/PXD021915] for PARP10 pull-down and PXD021916 [http://www.ebi.ac.uk/pride/archive/projects/PXD021916] for phosphoproteomics experiment. The source data underlying Figs. 2a-f, 3a-c, 4a-b, 5a-b and Supplementary Figs. 2a-e, 3a-d, 4a-c, 4e, 6, 7 and 8 are provided as a

Source Data file. Figs. 2-5 and Supplementary Figs. 2-8 are associated with the raw data. UniProt (https://www.uniprot.org/) and phosphositeplus (https://www.phosphosite.org/homeAction.action) databases were used in this paper. The ATP-binding proteins were matched to the Supplementary Data 3 in https://doi.org/10.1038/s41467-019-09107-y.

Field-specific reporting

Please select the one below that is the best fit for your research. If you are not sure, read the appropriate sections before making your selection.

- [x] Life sciences
- [ ] Behavioural & social sciences
- [ ] Ecological, evolutionary & environmental sciences

For a reference copy of the document with all sections, see nature.com/documents/nr-reporting-summary-flat.pdf

Life sciences study design

All studies must disclose on these points even when the disclosure is negative.

Sample size	No statistical methods were used to predetermine sample size. Appropriate power for discovery of true positive events was gained from at least three biological replicates and sample sizes were chosen based on similar studies published before.
Data exclusions	No data were excluded from the analyses presented in this paper.
Replication	All SIESTA findings were replicated in independent duplicate experiments, as detailed for each individual experiment in figure captions, data descriptions, and online methods. The other types of analyses were performed in at least 3 replicates. Conclusions were drawn from reproducible effects in all replicates of the data sets. All attempts at replication were successful.
Randomization	This is not relevant in this study, as our study utilizes biological replicates of lysates form cell lines or recombinant proteins, with no covariates such as patient to patient variability.
Blinding	Blinding was not performed in this study. This is not relevant to our study, since data was analyzed using unbiased statistics and no covariates exist that could be analyzed with a certain bias.

Reporting for specific materials, systems and methods

We require information from authors about some types of materials, experimental systems and methods used in many studies. Here, indicate whether each material, system or method listed is relevant to your study. If you are not sure if a list item applies to your research, read the appropriate section before selecting a response.

Materials & experimental systems

n/a	Involved in the study	
x		Antibodies
	x	Eukaryotic cell lines
x		Palaeontology and archaeology
x		Animals and other organisms
x		Human research participants
x		Clinical data
x		Dual use research of concern

Methods

n/a	Involved in the study	
x		ChIP-seq
x		Flow cytometry
x		MRI-based neuroimaging

Eukaryotic cell lines

Policy information about cell lines

Cell line source(s)	HCT116 and HELA from ATCC, USA
Authentication	No authentication was performed, as passage number 2 cells were thawed and used from the ATCC source.
Mycoplasma contamination	Cells were negative for mycoplasma, as tested by PCR.
Commonly misidentified lines (See ICLAC register)	No misidentified cell lines used.
